# Rhein, a Natural Anthraquinone Derivative, Attenuates the Activation of Pancreatic Stellate Cells and Ameliorates Pancreatic Fibrosis in Mice with Experimental Chronic Pancreatitis

**DOI:** 10.1371/journal.pone.0082201

**Published:** 2013-12-03

**Authors:** Siu Wai Tsang, Hongjie Zhang, Chengyuan Lin, Haitao Xiao, Michael Wong, Hongcai Shang, Zhi-Jun Yang, Aiping Lu, Ken Kin-Lam Yung, Zhaoxiang Bian

**Affiliations:** 1 School of Chinese Medicine, Hong Kong Baptist University, Kowloon Tong, Hong Kong SAR, China; 2 Tianjin University of Traditional Chinese Medicine, Tianjin, China; 3 Department of Biology, Hong Kong Baptist University, Kowloon Tong, Hong Kong SAR, China; Klinikum rechts der Isar der TU München, Germany

## Abstract

Pancreatic fibrosis, a prominent histopathological feature of chronic pancreatitis (CP) and pancreatic ductal adenocarcinoma, is essentially a dynamic process that leads to irreversible scarring of parenchymal tissues of the pancreas. Though the exact mechanisms of its initiation and development are poorly understood, recent studies suggested that the activation of pancreatic stellate cells (PSCs) plays a critical role in eliciting such active course of fibrogenesis. Anthraquinone compounds possess anti-inflammatory bioactivities whereas its natural derivative rhein has been shown to effectively reduce tissue edema and free-radical production in rat models of inflammatory conditions. Apart from its anti-inflammatory properties, rhein actually exerts strong anti-fibrotic effects in our current *in-vivo* and *in-vitro* experiments. In the mouse model of cerulein-induced CP, prolonged administration of rhein at 50 mg/kg/day significantly decreased immunoreactivities of the principal fibrotic activators alpha-smooth muscle actin (α-SMA) and transforming growth factor-beta (TGF-β) on pancreatic sections implicating the activation of PSCs, which is the central tread to fibrogenesis, was attenuated. Consequently, the overwhelmed deposition of extracellular matrix proteins fibronectin 1 (FN1) and type I collagen (COL I-α1) in exocrine parenchyma was found accordingly reduced. In addition, the expression levels of sonic hedgehog (SHH), which plays important roles in molecular modulation of various fibrotic processes, and its immediate effector GLI1 in pancreatic tissues were positively correlated to the degree of cerulein-induced fibrosis. Such up-regulation of SHH signaling was restrained in rhein-treated CP mice. In cultured PSCs, we demonstrated that the expression levels of TGF-β-stimulated fibrogenic markers including α-SMA, FN1 and COL I-α1 as well as SHH were all notably suppressed by the application of rhein at 10 μM. The present study firstly reported that rhein attenuates PSC activation and suppresses SHH/GLI1 signaling in pancreatic fibrosis. With strong anti-fibrotic effects provided, rhein can be a potential remedy for fibrotic and/or PSC-related pathologies in the pancreas.

## Introduction

Pancreatic fibrosis, a characteristic histopathological feature of chronic inflammation and carcinogenesis of the pancreas, is no longer being considered merely as an epiphenomenon of the diseases since it is actually an active dynamic process that results in overwhelmed production of fibrotic factors, imbalanced deposition of extracellular matrix (ECM) proteins and destructive scarring of the pancreatic parenchyma [1]. The progressive fibrotic cascade can eventually lead to the loss of pancreatic functions, systemic complications including hypercalcaemia, malabsorption and diabetes mellitus and/or tumor desmoplasia [2]. Pancreatic fibrosis is commonly arisen from inflammation, ductal obstruction or tissue injury [3]; however, its etiology can also be idiopathic or congenital, for instance, mutations in the *CFTR* gene [4]. A number of recent studies suggested that the most crucial step of the progression of fibrosis in the parenchymal tissue is the activation of pancreatic stellate cells (PSCs) [5,6] and which can be identified by the presence of the intermediate fibrotic filament alpha-smooth muscle actin (α-SMA or Acta2). 

PSCs comprising 4 to 7 % of all parenchymal cells are localized in the periacinar region of the exocrine pancreas [7]. Properties of stellate cells in the pancreas are similar to those present in other organs such as liver, kidney and lung. In normal condition, stellate cells are quiescent and can be detected by the autofluorescence of vitamin A accumulating in the cytoplasmic fat-droplets. Once the tissue is injured or inflamed, these resident stellate cells tend to lose their fat-droplets and transform into myofibroblast-like phenotype followed by the formation of fibrotic stress fibers; such transformation process is so-called “activation” [8]. In the case of pancreatic fibrogenesis, activated PSCs then express high levels of α-SMA and ECM proteins [9]. The course of myofibroblast-transformation is actually triggered by a complicated interplay among a variety of pro-fibrotic and pro-inflammatory mediators such as transforming growth factor-beta (TGF-β), platelet-derived growth factor (PDGF), tumor necrosis factor-alpha (TNF-α) and interleukin-1 beta (IL-1β) that are massively produced in a paracrine fashion in response to tissue injury or the elicitation of inflammation [10,11]. Meanwhile, activated PSCs secrete autocrine factors namely TGF-β in order to perpetuate the activating phenotype. Once the fibrotic signaling cascade is initiated, ECM proteins specifically fibronectin 1 (FN1) and type I collagen (COL I-α1) are deposited in the exocrine pancreas in large amounts for the purpose of tissue repair and/or regeneration so that inflammatory infiltrates can be replaced [1,11]. Most importantly, TGF-β is the pivotal activator involved in nearly all kinds of fibrotic conditions including hepatic fibrosis, pulmonary fibrosis and pancreatic fibrosis [12]. In fact, the initial events and the composition of fibrotic scarring are relatively common irrespective of the cause of injury or type of tissue. During the progression of fibrogenesis, myofibroblasts are capable of producing specialized enzymes including matrix metalloproteinases (MMPs) in order to facilitate the process of tissue repair [13]. MMPs are endopeptidases responsible for maintaining the balance between synthesis and degradation of ECM proteins. Reduced production of MMPs by PSCs impairs the extracellular proteolysis of ECM, and thus helps promote or sustain the fibrotic phenotype. Some recent studies suggested that by producing high levels of cytokines, growth factors and ECM proteins, PSCs even promote the initiation, development, metastasis and resistance to chemoradiation of pancreatic ductal adenocarcinoma (PDAC), which is a malignant tumor with a 5-year survival rate of less than 5 % [14,15]. For that reason, the activation of PSCs is indicative of the extent of fibrosis in the pancreas and in turn to be a logical target for therapeutic intervention of pancreatic fibrotic conditions and tumors. 

Hedgehog (HH) signaling is normally activated in inflammatory and fibrotic diseases such as systemic sclerosis, interstitial pneumonitis and injury-related inflammations [16–18]. Among the three HH homologues (Sonic-, Desert- and Indian-HH), sonic hedgehog (SHH) signals are suggested to play important roles in the pathogenesis of fibrosis [19,20]. Accumulating evidence indicates cross talks between SHH pathway and TGF-β signaling in a number of diseased conditions including gastric carcinoma [21], melanoma bone metastasis [22] and pulmonary fibrosis [20] that are independent of patched-1 (PTCH-1)- or smoothened (SMO)-inhibition in which PTCH-1 and SMO are the two major membrane spanning receptors for SHH. On the other hand, the three glioma-associated oncogene family zinc-finger members, GLI1, GLI2 and GLI3, which are requisite downstream components of the SHH pathway, are responsible for mediating the transcription of HH target genes in fibrotic conditions wherein GLI3, however, is rarely involved [23,24]. HH inhibitor LDE223 has been manifested to provide effective anti-fibrotic effects in a mouse model of systemic sclerosis by suppressing the activation of *Gli* transcription factors [25]. Hence, the suppression of SHH signaling may play a crucial role in the modulation of pro-fibrotic factors in pancreatic fibrogenesis.

**Figure 1:**
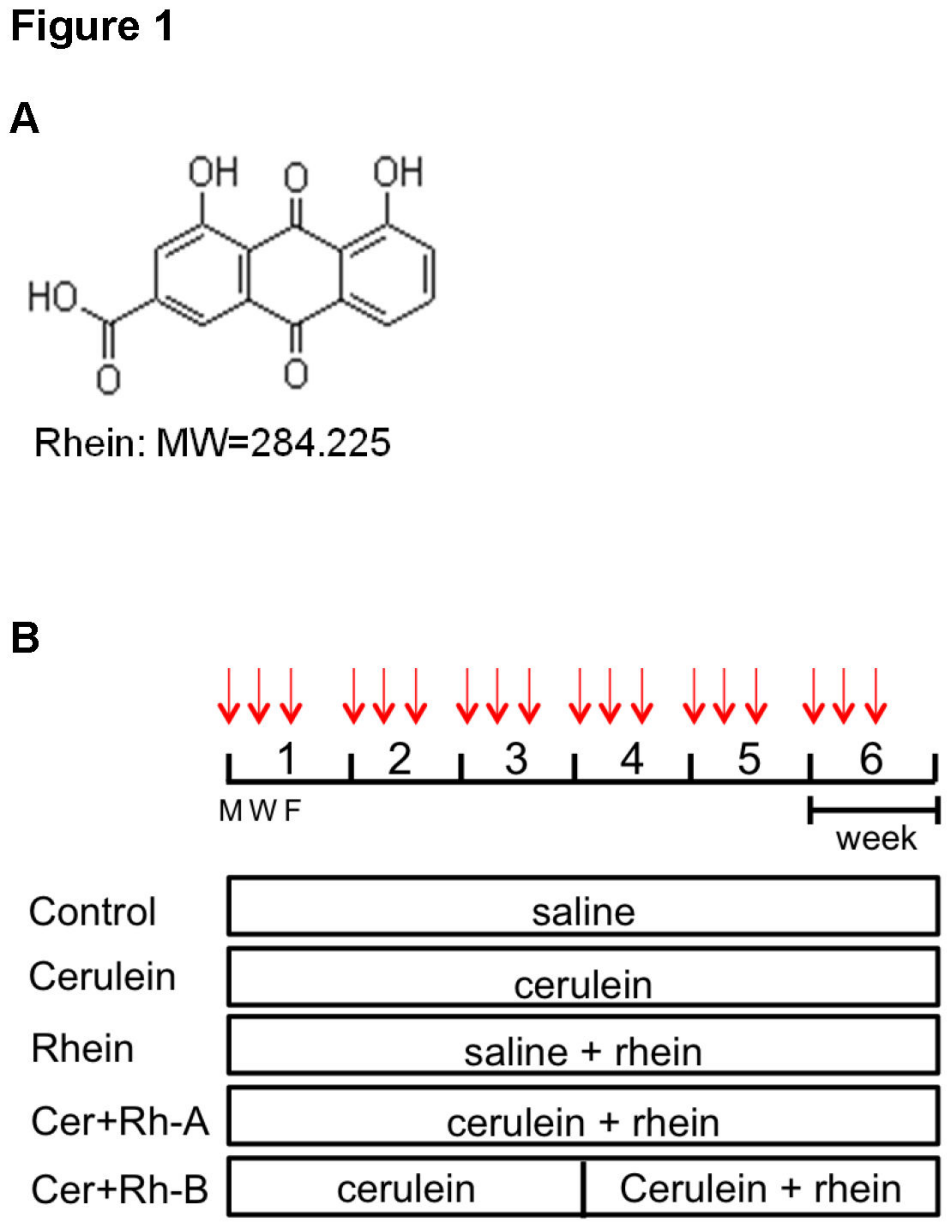
Compound used and experimental design. (A) The chemical structure of rhein. (B) Schematic chart showing the experimental timeline of induction of CP with cerulein. Mice were randomly divided into 5 groups (n=8). Control group received only i.p. injections of saline whereas Cerulein group received i.p. injections of cerulein at 50 μg/kg 3 days a week in addition to the oral administration of 0.5 % NaCMC. Rhein group was given with i.p. injections of saline and oral gavage of rhein at 50 mg/kg/day. Cer+Rh-A group was given with i.p. injections of cerulein plus oral administration of rhein from the first day of experimental period till the end in a total of 6 weeks whereas Cer+Rh-B group received same cerulein injections as Cer+Rh-A but with rhein treatment from week 4 till the end of experimental period in a total of 3 weeks.

Rhein is a natural anthraquinone derivative, also known as 9, 10-Dihydro-4, 5-dihydroxy-9, 10-dioxo-2-anthracenecarboxylic acid (MW=284.225; Figure 1A), that can be extracted from roots of Polygonaceae (rhubarb). This yellow crystalline rhubarb extract has been serving as a mild laxative agent as well as an astringent since ancient times in the Chinese population [26]. In recent decades, administration of rhein in the range of 25 to 100 mg/kg/day has been demonstrated to exert diverse pharmacological actions including anti-microbial [27], anti-angiogenic [28] and anti-cancer activities [29,30]. In some latest reports, Cong et al showed that the anti-inflammatory effect of rhein was actually comparable to the accredited pain-killer ibuprofen in adjuvant-induced inflammation via a significant amelioration of oxidative stress [31] whereas Fernand and colleagues claimed that rhein provided anti-angiogenic effect against breast cancer cell proliferation and migration by inactivating the phosphatidylinositol 3-kinase pathway [32]. The underlying mechanisms for anthraquinone derivatives have yet been relatively delineated; at least involve the partial inhibition of cyclooxygenase 2 [33] and the negative modulation the p38 MAPK cascade [34]. Interestingly, a relevant article from He et al revealed that rhein effectively blocked the activation of renal interstitial fibroblasts in mice [35] whereas a study from Guo et al reported that rhein at a dose as low as 25 mg/kg/day provided significant protective effect against carbon tetrachloride-induced liver fibrosis in rats [36]. Taken together, this anthraquinone derivative appears to be a potential remedy not only for the treatment of inflammatory diseases, but also for fibrogenic events; therefore, the usage of rhein in pancreatic fibrosis urges a thorough investigation.

In the present study, we hypothesize that rhein, as a natural anti-fibrotic compound, attenuates the activation of PSCs and ameliorates the severity of pancreatic fibrosis in experimental chronic pancreatitis (CP). Therefore, we evaluated the anti-fibrotic effects of rhein *in-vivo* and *in-vitro* respectively in a mouse model of cerulein-induced CP and in a cellular model of immortalized rat PSCs. Systemic histopathological, immunohistochemical and biochemical analyses of CP and cellular fibrogenesis were performed.

## Materials and Methods

### Animals and experimental design

Male C57/BL6 mice (20 to 24 g) at an age of 6 to 8 weeks obtained from the Chinese University of Hong Kong (Hong Kong SAR, China) were housed in standard cages in a humidity-controlled room with an ambient temperature of 23 ± 2 °C and 12 h light/dark cycle. They were fed with standard laboratory chow, given water *ad libitum* and randomly assigned into 5 groups of 8 animals each according to Figure 1B. In brief, for the induction of CP, Cerulein group received 6 intra-peritoneal (i.p.) injections of cerulein (Sigma) at supra-maximal dose of 50 μg/kg 3 days a week and was daily fed with 0.5 % sodium carboxymethyl cellulose (NaCMC, Sigma) in a volume of 0.4 mL; Control group received same number of injections of saline and daily oral gavage of 0.5 % NaCMC; Rhein group was also given with saline injections but fed with rhein (purity ≥ 98 %, Nanjing Zelang Medical, China) dissolved in 0.5 % NaCMC by gavage at 50 mg/kg/day; Cer+Rh-A group received cerulein injections plus rhein treatment at 50 mg/kg/day from the 1st day of experimental period till the end in a total of 6 weeks mimicking a prophylactic course of treatment; Cer+Rh-B group received cerulein injections same as the Cer+Rh-A group but was given with rhein starting from the 1st day of week 4 till the end of experiment in a total of 3 weeks mimicking a therapeutic course of treatment. All animals were sacrificed at the end of the 6-week study period. Ethical approval for all husbandry and experimental procedures had been obtained from the Committee on the Use of Human and Animal Subjects in Teaching and Research (HASC) of Hong Kong Baptist University and was in accordance with the Animals Ordinance, Department of Health, Hong Kong SAR, China.

### Histological examination

Upon scarification, pancreata were immediately removed, trimmed from fat, weighed and fixed in 4 % paraformaldehyde-phosphate buffered saline (PBS) overnight at 4 °C. Samples were then processed, embedded in paraffin wax and sectioned. Hematoxylin-eosin (H & E) staining was performed according to standard procedures and images were captured under Nikon light microscope equipped with the SPOT Advanced program. Histopathological assessment of CP was scored based on integrity of pancreatic architecture, infiltration of immune cells and glandular atrophy on the H&E staining sections. Abnormal pancreatic architecture was identified by non-intact exocrine acini, loss of lobular structure and enlarged interstitial spaces; inflammatory cell infiltration was identified by deposition of immune cells scattered across the tissue section; glandular atrophy was identified by the loss of pancreatic mass as well as the population sizes of acini.

### Cell line and culture condition

Rat pancreatic stellate cell line LTC-14 [37] was kindly provided by Prof. Robert Jaster from University Hospital of Rostock, Germany and routinely maintained in IMDM (GIBCO) supplemented with 10 % fetal bovine serum (FBS, GIBCO), 1 % penicillin-streptomycin in a 5 % CO_2_, 95 % air humidified atmosphere at 37 °C. Recombinant TGF-β, TNF-α and SHH utilized in the *in vitro* experiments were all purchased from Sigma-Aldrich.

### Immunofluorescent staining

Paraffin-embedded pancreatic samples were deparaffinized and rehydrated according to standard procedures. Sections were subjected to the antigen retrieval process using 0.1 N citric buffer prior to the blocking step. LTC-14 cells were seeded at a density of 1 × 10^5^ onto poly-L-lysine-coated cover slips in 24-well plates, pre-incubated with or without recombinant TGF-β (5 ng/mL) plus rhein (0 to 100 μM) in serum-free medium (SFM) for 24 hours. The cells were subsequently washed with PBS and fixed in acetone:methanol (1:1, v/v) for 20 min. After rinsing, tissue sections or fixed cells were blocked with 3 % bovine serum albumin (BSA), incubated with primary antibodies against α-SMA (Abcam), SHH or GLI1 (Santa Cruz Biotechnology) overnight at 4 °C, and detected using FITC- or Rhodamine-conjugated anti-rabbit or anti-goat secondary antibodies. Slides were then mounted with fluorescence mounting medium containing 4’, 6-diamidino-2-phenylindole (DAPI, Sigma-Aldrich). Images were captured using the Nikon microscope and analyzed with the SPOT Advanced software.

### Sirius Red and Fast Green staining

The amounts of collagen and non-collagenous proteins in pancreatic sections were determined using Sirius Red/Fast Green collagen staining kit (Chondrex Inc.) according to the manufacturer’s instruction. In brief, collagen proteins in pancreatic sections were stained with Sirius Red whereas non-collagenous proteins were stained with Fast Green. With the addition of dye extraction solution, color was eluted from the tissue sections. Absorbance at 540 nm and 640 nm were taken respectively for the amount of collagen and non-collagenous proteins. Data was expressed as a ratio of collagen proteins to total proteins.

### Cell viability assay

LTC-14 cells were seeded in 96-well plates at a density of 5 × 10^3^, serum-starved overnight, and subsequently treated with a serial concentrations of rhein (0.1 to 200 μM) in SFM for 24 hours. Control cells were treated with 0.1 % dimethyl sulfoxide (DMSO) in IMDM as rhein powder was dissolved in DMSO. The cytotoxicity of rhein was evaluated by incubating the treated cells with MTT reagent at 37 °C for 3 hours and then with isopropanol-HCl at room temperature for 0.5 hour. Spectrophotometric absorbance of the samples was measured at 570 nm using a microplate reader (Bio-rad).

### Real-time quantitative PCR (qPCR)

Total RNA was extracted from pancreatic tissues or LTC-14 cells using TRIzol reagent (Invitrogen) according to the manufacturer’s instruction. Two μg of total RNA of each sample was then transcribed into cDNA using Supercriptase III (Invitrogen) in a total volume of 20 μL. The synthesized cDNA was applied to amplifications with mouse-specific or rat-specific primers for *Tgf-β1*, *Acta2*, *Col I-α1*, Fn*1*, *Mmp2*, *Shh*, *Gli1 Gli2* and *Gapdh* for 40 cycles in the ABI ViiA 7 real-time PCR system (Applied Biosystems) using 2X SYBR Green PCR Master Mix (Applied Biosystems). Expression of gene of interest of each sample was normalized to the endogenous control *Gapdh*. Fold changes were calculated using the comparative CT (2^-ΔΔCT^) method. Primer sequences for qPCR analysis will be furnished upon request.

### Western blot analysis

Total protein was extracted from pancreatic tissues homogenized with homogenization buffer or from LTC-14 cells lysed with RIPA buffer containing protease inhibitors. For detection of NF-κB, nuclear fraction was used. Tissue extracts or cell lysates were loaded, separated by 10 to 15 % SDS-polyacrylamide gel electrophoresis, and transferred onto PVDF membranes (Bio-rad) by wet electroblotting. The membranes were blocked with 5 % non-fat dry milk in Tris-buffered saline containing 0.1 % Tween 20 (TBST) for 1 h at room temperature, incubated with anti-α-SMA (Abcam), FN1 (Novous), NF-κB p65, phospho-p-38 MAPK (Cell signaling), SHH, IκB-α, β-ACTIN or LAMININ (Santa Cruz Biotechnology) antibodies overnight at 4 °C, and subsequently incubated with corresponsive horseradish peroxidase-conjugated anti-rabbit, anti-goat or anti-mouse secondary antibodies. Proteins were eventually visualized by utilization of an ECL kit (GE Healthcare) and normalized to the expression level of β-ACTIN or LAMININ.

### Depletion of SHH by small interfering RNA (siRNA) in LTC-14 cells

siRNA duplex specifically targeting rat *Shh* (sense: 5’-GCC GAU AUG AAG GGA AGA U-3’; anti-sense: 5’-AUC UUC CCU UCA UAU CGG C-3’) and a non-silencing control duplex (sense: 5’-GCC AUG UAA GGA GAG AGA U-3’; anti-sense: 5’-AUC UCU CUC CUU ACA UGG C-3’) were purchased from Invitrogen. Twenty-four hours before transfection, 1 × 10^5^ cells were seeded per well in 24-well plates in 0.5 mL complete culture medium without antibiotics. The siRNA oligos at 20 nM were delivered into LTC-14 cells using Lipofectamine 2000 (Invitrogen). The silencing effects were examined by means of qPCR and Western blotting.

### Cytokine and amylase assays

Levels of amylase, TNF-α and IL-1β in animal serum samples were determined using commercial amylase kits (Pointe, USA) or cytokine ELISA kits (eBioscience, USA) respectively according to manufacturer’s instructions. After the termination of HRP reactions with 2 M sulfuric acid, optical densities of the samples were taken using a microplate reader at 405 nm. Data were expressed as pg/mL per 25 μl of serum.

### Statistical analysis

The statistical differences were determined using one-way analysis of variance (ANOVA) followed by Tukey’s test as a *post hoc* test. All values were expressed as means ± standard derivation (SD). *P* value of < 0.05 was accepted as statistically significant.

## Results

### Rhein ameliorates cerulein-induced chronic pancreatitis

**Figure 2:**
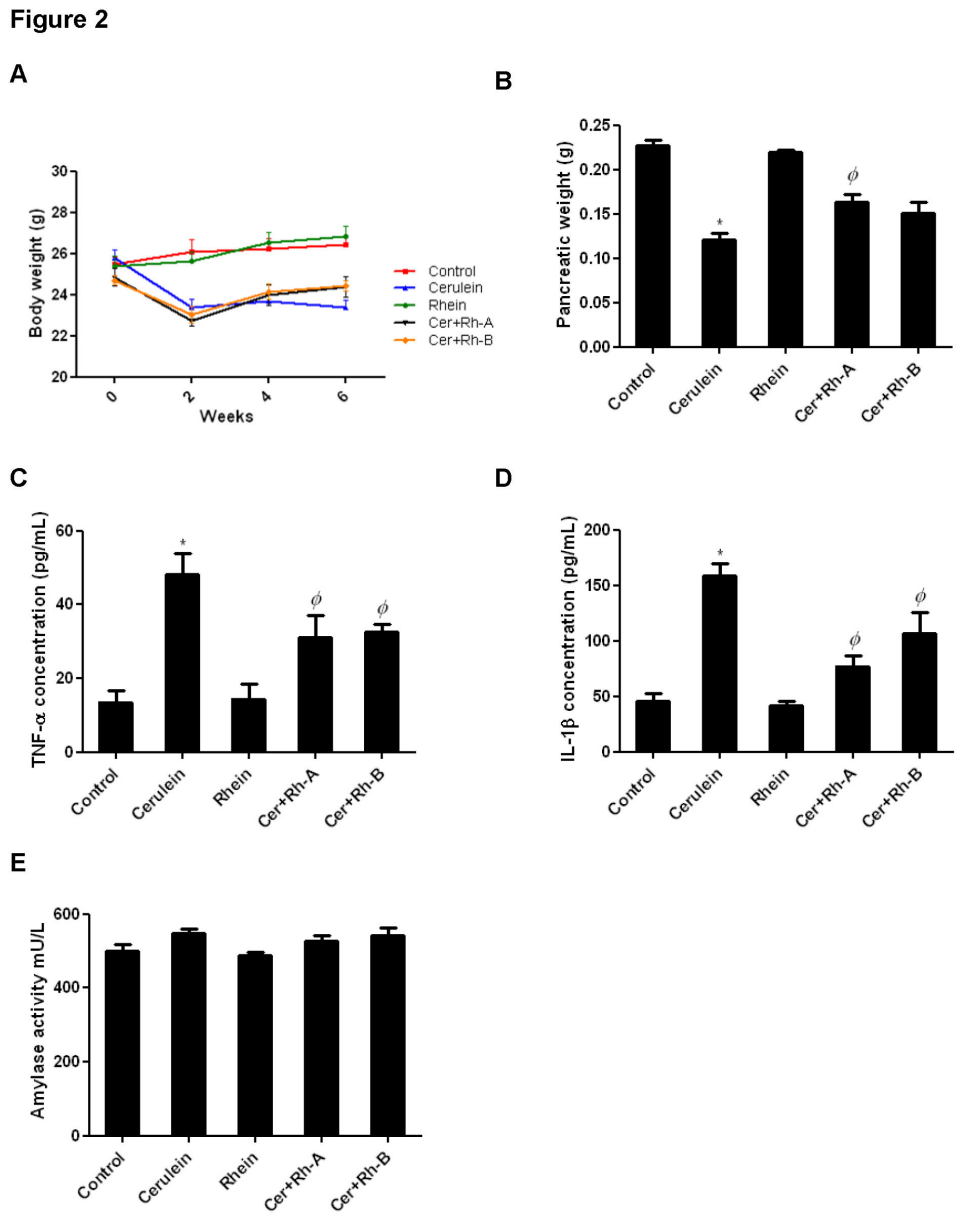
Assessment of CP in mice. (A) Changes of body weight among the 5 groups of mice in experimental period of 6 weeks. Animals were weighed every other day over the 6-week period. (B) Wet weights of pancreatic tissues were measured at the time of sacrifice. (C, D) By means of ELISA, serum levels of TNF-α and IL-1β in mice were measured at the end of the 6-week experiment and were expressed as pg/mL. * *p*< 0.05 when comparing to the Control group whereas *Φ* *p*< 0.05 when comparing to the Cerulein group. (E) Activities of α-amylase in sera were expressed as mU/L and found statistically unchanged among all groups.

**Figure 3:**
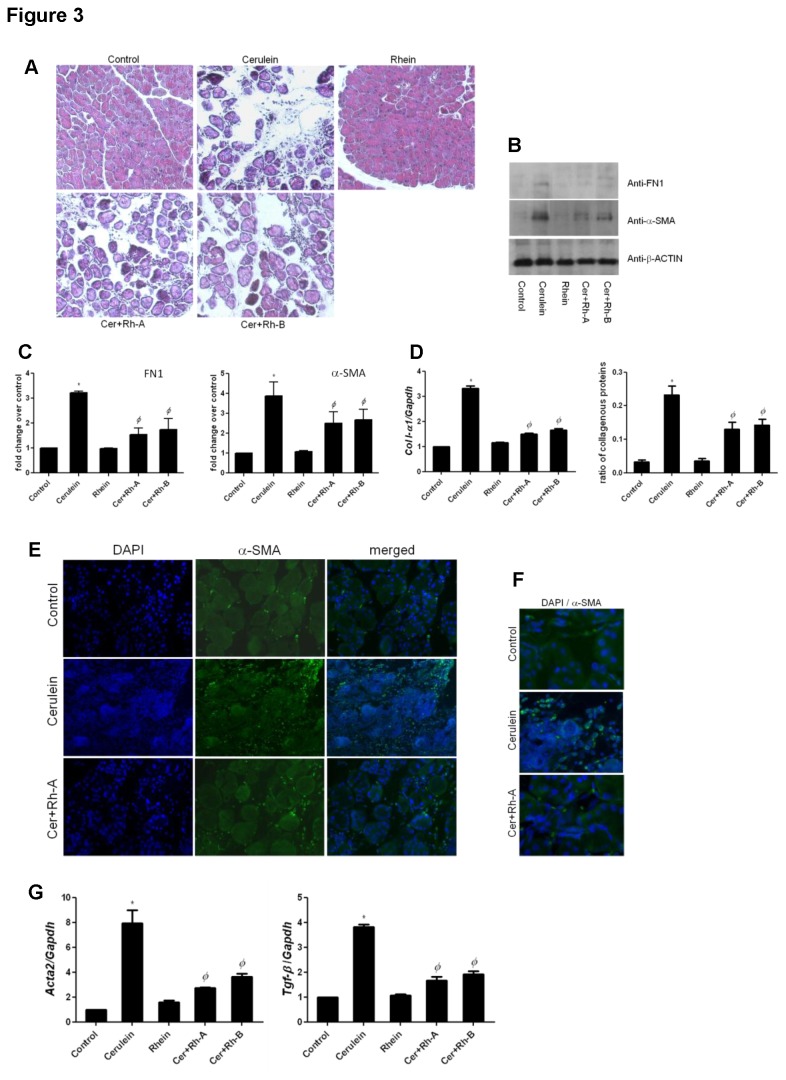
Rhein ameliorates the severity of pancreatic fibrosis and CP. (A) Pancreatic tissues collected from the 5 experimental groups were subjected to standard H&E staining. Abnormal architecture, glandular atrophy and inflammatory cell infiltrates were evaluated from the H&E sections. Magnification 200 ×. (B) By means of Western blotting analyses, the expression levels of α-SMA and FN1 in pancreatic homogenates were examined. (C) Integrated densities of the immunobands of α-SMA and FN1 were measured, normalized to the loading reference β-ACTIN and calculated as fold changes over the Control group. Readings were taken from 4 individual blots. (D) Synthesis of collagen I-α1 mRNA and protein was determined by means of qPCR and Sirius Red staining respectively. Collagen proteins were stained with Sirius Red whereas non-collagenous proteins were stained with Fast Green. Color was eluted from the tissue sections and measured at 540 nm and 640 nm. * *p*< 0.05 when comparing to the Control group whereas *Φp*< 0.05 when comparing to the Cerulein group. (E) In paraffin-embedded pancreatic tissues, the activated PSCs were stained green (FITC) with α-SMA-antibody whereas nuclei were stained blue with DAPI. Magnification 200 ×. (F) Images enlarged from (E) using the SPOT Advanced program for a more detailed view of the activated PSCs. (G) mRNA expression levels of *Acta2* and *Tgf-β* in pancreatic tissues were determined by means of qPCR using SYBR Green reagent, normalized to the internal reference *Gapdh* and expressed as fold changes over the Control group. * *p*< 0.05 when comparing to the Control group whereas *Φ* *p*< 0.05 when comparing to the Cerulein group.

Rhein had no adverse or harmful effects in animals as no significant alterations were found between the Control group and the Rhein only group in the aspect of serum levels of TNF-α, IL-1β and amylase, histology of pancreatic tissues as well as body weights (Figures 2A-E). In the Cerulein group, a successfully induced CP model was reflected by severe pancreatic damages including abnormal architecture, glandular atrophy, enlarged interstitial spaces and inflammatory cell infiltrates in pancreatic sections stained by H&E (Figure 3A) and a notable loss of body weight (Figure 2A). With the administration of rhein for 6 weeks (Cer+Rh-A group) and 3 weeks (Cer+Rh-B group) for prophylactic and therapeutic treatments respectively, CP-induced tissue damages in terms of pancreatic architecture were markedly alleviated (Figure 3A). The overall histopathological scores of CP, in terms of glandular atrophy, inflammation and fibrosis, for all 5 groups of animals were summarized in Table 1. Severe loss of pancreatic acini or glandular atrophy was assessed by an obvious decline in the wet weight of the tissue in Cerulein group (0.1206 g ± 0.01570) when comparing to the Control group (0.2272 g ± 0.01116). In groups Cer+Rh-A and Cer+Rh-B, pancreatic weights were significantly restored by 35 % (0.1635 g ± 0.01913) and 25 % (0.1510 g ± 0.02879) respectively when comparing to the Cerulein group (Figure 2B). Serum levels of pro-inflammatory cytokines TNF-α and IL-1β were elevated approximately 2.5 folds under CP condition (TNF-α in Cerulein group: 48.10 pg/mL ± 9.877 vs. TNF-α in Control group: 13.52 pg/mL ± 5.575); however, such systemic augmentations of cytokines were reduced by 35 to 40 % in rhein-treated CP mice (TNF-α in Cer+Rh-A group: 31.18 pg/mL ± 10.45; TNF-α in Cer+Rh-B group: 32.52 pg/mL ± 3.761) when comparing to those of the Cerulein group (Figures 2C and 2D). Nevertheless, the serum α-amylase levels were not statistically affected by the induction of CP with cerulein or the treatment with rhein (Figure 2E).

**Table 1:** The histological scorings of clinical parameters for assessing the severity of CP.

	**Control**	**Cerulein**	**Rhein**	**Cer+Rh-A**	**Cer+Rh-B**
**Glandular atrophy**	0 ± 0	3.00 ± 0.41 *	0 ± 0	1.88 ± 0.49 #	2.00 ± 0.41 #
**Fibrosis**	0 ± 0	2.25 ± 0.29 *	0 ± 0	1.13 ± 0.25 #	1.25 ± 0.29 #
**Inflammation**	0 ± 0	2.75 ± 0.65 *	0 ± 0	1.38 ± 0.48 #	1.38 ± 0.25 #
**Damage Index**	0 ± 0	8.00 ± 0.91 *	0 ± 0	4.38 ± 0.25 #	4.63 ± 0.48 #

* *p*< 0.05 when comparing to the Control group whereas # *p*< 0.05 when comparing to the Cerulein group.

### Rhein reduces fibrosis in pancreatic tissues

FN1 and α-SMA are two major fibrosis indicators for ECM deposition and degree of fibrosis. Western blotting results revealed that the expression levels of FN1 and α-SMA were up-regulated correspondingly by approximately 3 and 4 folds in cerulein-treated CP mice when comparing to those in the Control group and such up-regulations were significantly suppressed by roughly 40 % in both Cer+Rh-A and Cer+Rh-B groups (Figure 3B). Densitometries of the immunobands of each group were quantified, normalized to the loading reference and calculated as fold change over the Control group (Figure 3C). By means of qPCR, mRNA level of *Col I-α1*, another crucial fibrotic ECM marker, was found with a 3-fold increase in CP pancreas when comparing to the control; meanwhile, the elevated synthesis of *Col I-α1* was repressed by more than 50 % in both Cer+Rh-A and Cer+Rh-B groups (Figure 3D). The deposition of collagen, expressed as a ratio of Sirius Red (collagen-positive proteins)/Fast Green (collagen-negative proteins) staining, was detected with a 6-fold increase in the cerulein-induced pancreatic sections (0.2325 ± 0.0350) when comparing to that in the Control group (0.0325 ± 0.01258), and such increase was significantly reduced by nearly 40 % in the CP groups with prophylactic (0.130 ± 0.04163) and therapeutic (0.1425 ± 0.03594) treatments of rhein (Figure 3D). Immunohistochemistry (IHC) of α-SMA staining was performed in order to quantify the activated PSCs in pancreatic tissues of the animals. The intensities of positive immunofluorescent signals of α-SMA were increased from 0.0214 % in control pancreas to 0.678 % of the captured areas in pancreatic sections of cerulein-treated mice, and such intensities were remarkably diminished to 0.119 % in the mice of Cer+Rh-A group that was fed with rhein (Figure 3E). The zoomed images provided more comprehensible views of the localization of activated PSCs on the pancreatic sections as represented by α-SMA-immunoreactivities, and the fluorescent signals of α-SMA were obviously reduced by the rhein treatment (Figure 3F). Further, the pivotal fibrogenic activator TGF-β was also examined in this study. Similar to *Acta2*, the mRNA level of *Tgf-β* was drastically up-regulated by 4 folds in the Cerulein group when comparing to the Control group and the cerulein-induced elevation was reduced by 2 folds in the rhein-treated CP groups (Figure 3G).

### Rhein modulates Shh signaling via Gli1 in CP and fibrogenesis

**Figure 4:**
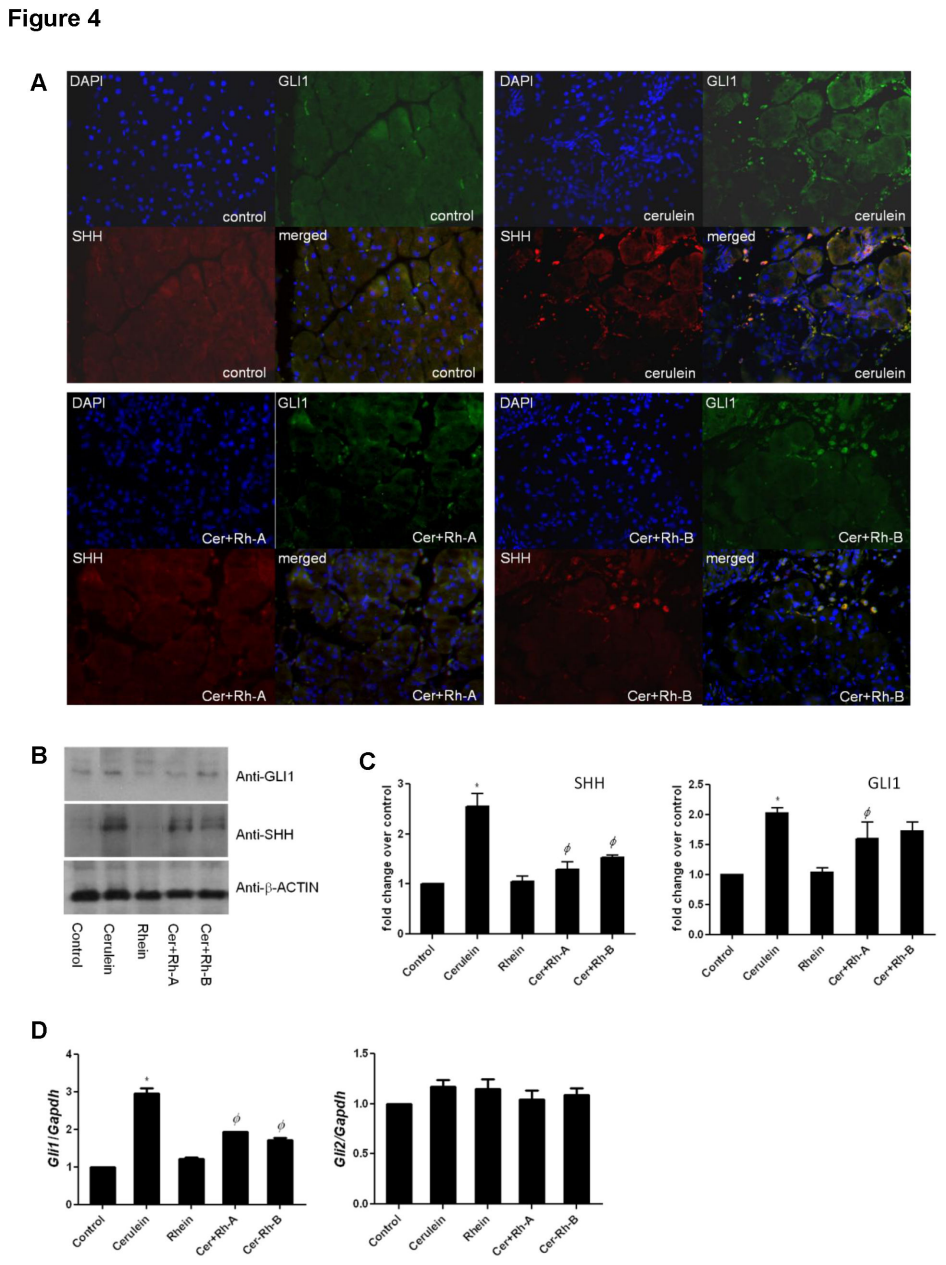
Rhein suppresses SHH signaling in-vivo. (A) The fluorescent images demonstrated the immunoreactivites of GLI1 (green; FITC) and SHH (red; Rhodamine) in paraffin-embedded pancreatic tissues in which nuclei were stained blue with DAPI. (B) Protein levels of GLI1 and SHH in pancreatic homogenates of the mice were visualized on immunoblots probed with anti-GLI1 and anti-SHH antibodies whereas β-ACTIN was served as the loading reference. (C) Integrated densities of the immunobands of SHH and GLI1 were measured. Readings were normalized to the internal reference β-ACTIN and expressed as fold change over control. (D) Transcripts of *Gli1* and *Gli2* were amplified by means of qPCR, normalized to the endogenous reference *Gapdh* and expressed as fold changes over the Control group. * *p*< 0.05 when comparing to the Control group whereas *Φ* *p*< 0.05 when comparing to the Cerulein group.

**Figure 5:**
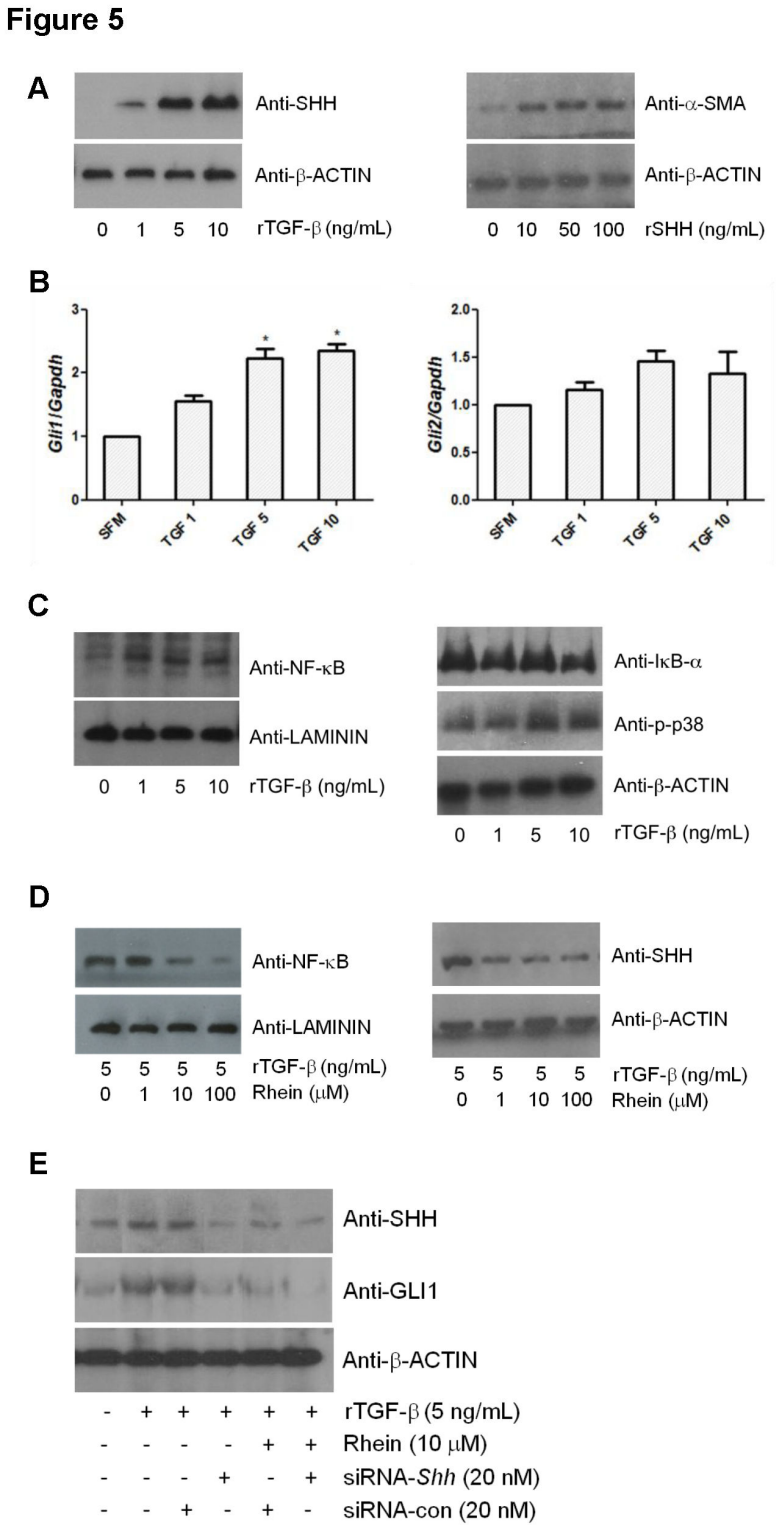
Rhein inhibits SHH and NF-κB signals *in-vitro*. (A) LTC-14 cells were treated with recombinant TGF-β (0, 1, 5 or 10 ng/mL) or SHH (0, 10, 50 or 100 ng/mL) in SFM for 24 hours and subjected to Western blotting analyses. Immunoblots were probed with anti-SHH antibody for the TGF-β treatment or probed with anti-α-SMA antibody for the SHH treatment. β-ACTIN was served as a loading reference. (B) LTC-14 cells were treated with TGF-β (0, 1, 5 or 10 ng/mL) in SFM for 24 hours and harvested for mRNA extraction. Transcripts of *Gli1* and *Gli2* were amplified by means of qPCR, normalized to the endogenous reference *Gapdh* and expressed as fold changes over the non-TGF-β-treated control (SFM control). * *p*< 0.05 when comparing to the SFM control. (C) LTC-14 cells were treated with TGF-β (0, 1, 5 and 10 ng/mL) in SFM for 24 hours and harvested for protein extraction. Immunoblots were probed with anti-NF-κB p65, IκB-α, p-p38, β-ACTIN and LAMININ antibodies. β-ACTIN and LAMININ were served as loading references for the cytoplasmic and nuclear extracts respectively. (D) LTC-14 cells were treated with TGF-β (5 ng/mL) and rhein (0, 1, 10 or 100 μM) in SFM for 24 hours and subjected to Western blotting analyses. (E) LTC-14 cells were transfected with siRNA duplex targeting *Shh* (20 nM) or a non-silencing control duplex (20 nM) for 24 hours and incubated with TGF-β (5 ng/mL) and/or rhein (10 μM) prior to protein extraction.

Shh signaling plays important roles in a number of cellular processes including fibrogenesis. By means of IHC staining, we found that the ratio of immunoreactivities of SHH/GLI1 to DAPI-stained nuclei was obviously enhanced under CP condition (16.58 % ± 3.4071) when comparing to that of the control (3.33 % ± 1.884). With the administration of rhein, the SHH/GLI1 immunoreactivities were suppressed remarkably to 8.65 % ± 2.1462 and 10.37 % ± 1.7954 in Cer+Rh-A and Cer+Rh-B respectively (Figure 4A). In agreement to the results of immunofluorescent staining, the up-regulation of SHH and GLI1 signals was also observed in the pancreatic homogenates of CP mice by means of Western blotting (Figure 4B). Densitometries of the immunobands of each group were quantified, normalized to the loading reference and calculated as fold change over the Control group (Figure 4C). The qPCR result further revealed that Shh signaling in CP was mediated through *Gli1* but not *Gli2* as *Gli1* transcript was subject to a 3-fold up-regulation whereas *Gli2* transcript remained unaffected (Figure 4D). Most importantly, the cerulein-induced elevations of SHH and GLI1 were repressed by 30 % in the pancreatic tissues of groups of rhein-treated CP mice (Figures 4B-D). In our *in-vitro* model, exogenous addition of recombinant TGF-β significantly increased the expression levels of SHH in a concentration-dependent fashion in LTC-14 cells. On the contrary, recombinant SHH up-regulated the expression of α-SMA in a manner comparable to TGF-β-stimulation (Figure 5A). Upon the challenge of TGF-β, merely the transcripts of SHH immediate downstream regulator *Gli1*, but not *Gli2*, were concentration-dependently increased in LTC-14 cells (Figure 5B). Meanwhile, expression levels of NF-κB in the nuclear extract and its downstream mediator p38 MAPK were as well elevated by TGF-β implicating that NF-κB signaling was mechanistically associated with the SHH pathway in the modulation of pro-fibrotic factors. In contrast to the elevation of NF-κB in the nuclear fraction, the expression of its inhibitory subunit IκB-α in the cytoplasm was decreased upon the challenge of TGF-β in LTC-14 cells (Figure 5C). In conclusion, rhein at 10 μM could significantly suppress the TGF-β-induced trans-activation of NF-κB and SHH signaling (Figure 5D). Further, our Western blotting results showed that the TGF-β-elevated expression levels of SHH and GLI1 were remarkably attenuated by approximately 70 % when siRNA duplex targeting *Shh* was applied to LTC-14 cells in which the inhibitory effects of rhein were diminished (Figure 5E). 

### Rhein attenuates fibrogenic mediators in response to pro-fibrotic agent *in-vitro*

**Figure 6:**
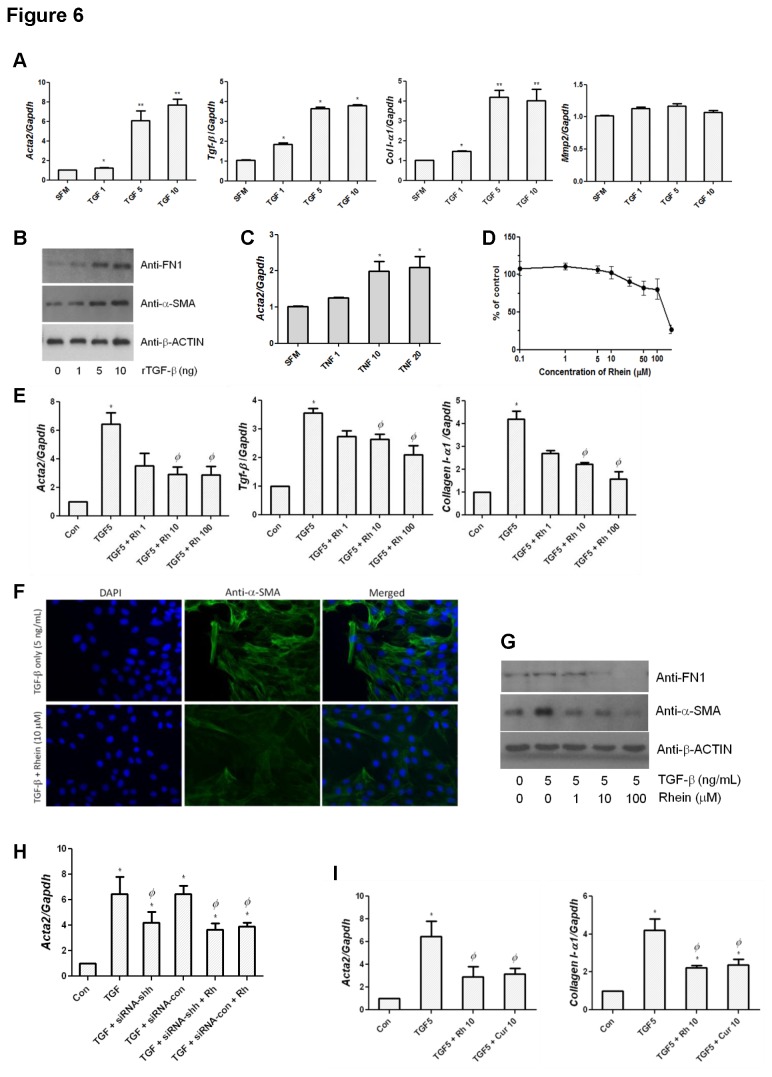
Rhein attenuates fibrotic mediators *in-vitro*. LTC-14 cells were treated with TGF-β (0, 1, 5 and 10 ng/mL) in SFM for 24 hours and harvested for mRNA extraction (A) or protein extraction (B). Transcripts of various fibrotic markers *Tgf-β*, *Acta2* and *Col* *I-α1* were amplified by means of qPCR using SYBR Green reagent, normalized to the endogenous reference *Gapdh* and expressed as fold changes over SFM control. * *p*< 0.05 and ** *p*< 0.01 when comparing to the non-TGF-β-treated SFM control. Changes of FN 1 and α-SMA proteins were determined by immunoblotting analyses. β-ACTIN was served as a loading reference. (C) LTC-14 cells were treated with TNF-α (0, 1, 10 and 20 ng/mL) in SFM for 24 hours and harvested for mRNA extraction. Expression level of *Acta2* was determined after normalizing to the endogenous reference *Gapdh*. * *p*< 0.05 when comparing to the SFM control. (D) Cytotoxicity of rhein was assessed using MTT assay. LTC-14 cells were treated with rhein at a series of concentrations for 24 hours and subjected to the MTT assay. The LD_50_ of rhein is approximately 120 μM. LTC-14 cells were pre-incubated with or without TGF-β (5 ng/mL) for 2 hours, treated with rhein (1, 10 and 100 μM) in SFM for 24 hours and harvested for mRNA extraction (E) or immunofluorescent staining (F) or protein extraction (G). By means of qPCR, expression levels of *Tgf-β*, *Acta2* and *Col* *I-α1* were determined. * *p*< 0.05 when comparing to the SFM control and *Φ* *p*< 0.05 when comparing to the TGF-β-treated control. Immunoreactivities of α-SMA were visualized by FITC (green) whereas nuclei were stained blue with DAPI. Magnification 400 ×. (H) LTC-14 cells were transfected with siRNA duplex targeting *Shh* (20 nM) or the non-silencing control duplex (20 μM) for 24 hours prior to the incubation of TGF-β (5 ng/mL) with or without treatment of rhein (10 μM). By means of qPCR, expression levels of *Acta2* were determined. (I) LTC-14 cells were pre-incubated with or without TGF-β (5 ng/mL) for 2 hours and treated with rhein (Rh) or curcumin (Cur) at 10 μM for 24 hours prior to mRNA extraction. By means of qPCR, expression levels of *Acta2* and *Col* *I-α1* were determined. * *p*< 0.05 when comparing to the SFM control and *Φ* *p*< 0.05 when comparing to the TGF-β-treated control.

As TGF-β is a pivotal pro-fibrotic mediator involved in nearly all fibrotic conditions, we tested its inducing effects on some important interplaying pro-fibrogenic factors in LTC-14 cells. By means of qPCR, we noticed that the challenge of recombinant TGF-β (1 to 10 ng/mL) concentration-dependently increased transcript levels of *Acta2*, Fn*1* and *Col I-α1*; however, *Mmp2*, which encodes a major endopeptidase for modulating ECM degradation, was unaffected (Figure 6A). Similar inductive effects of TGF-β were also observed on the expression levels of α-SMA and FN1 proteins on immunoblots (Figure 6B). Furthermore, LTC-14 cells also responded to the stimulation of recombinant TNF-α (1 to 20 ng/mL) and led to an augmentation of *Acta2* (Figure 6C). This piece of data implicated that pro-inflammatory cytokines induce pro-fibrotic events. Among a series of concentrations, TGF-β at 5 ng/mL was assessed to be a promising dose for a statistically significant induction of all the three major fibrogenic markers mentioned above. By means of MTT assay, the LD_50_ of rhein was found to be around 120 μM in LTC-14 cells (Figure 6D). Concentration at 10 μM was minimally required for a sufficient suppression of the TGF-β-induced up-regulation of fibrogenic mediators. Our qPCR results showed that TGF-β at 5 ng/mL provided a substantial elevation on mRNA expression levels of *Acta2*, Fn*1* and *Col I-α1* by approximately 3.5 to 6 folds and rhein at 10 μM significantly suppressed the TGF-β-induced elevations (Figure 6E). Similar inhibitory effect on α-SMA by rhein was also observed in immunofluorescent images (Figure 6F). Rhein also inhibited the expression levels of FN1 and α-SMA proteins in a dose-dependent manner as shown on Western blot images (Figure 6G) and such results were in accordance with qPCR and IHC analyses. Under *Shh*-depletion, the reductive effect of rhein on *Acta2* upon TGF-β-stimulation was obliterated in LTC-14 cells (Figure 6H). This piece of data further strengthened the involvement of Shh signaling in the activation of fibrotic mediators. Interestingly, our qPCR result demonstrated that the suppressive effects of rhein against fibrogenic mediators were actually comparable to the well known anti-fibrotic agent curcumin when both compounds were applied to LTC-14 cells at 10 μM (Figure 6I). Taken together, we conclude that rhein can effectively inhibit fibrotic mediators *in-vivo* and *in-vitro*. 

## Discussion

The natural anthraquinone compound rhein has been utilizing as a laxative agent since ancient times in the Chinese populations [26]. Recent studies reported that anthraquinone once undergoes metabolisms; it induces secretion of water and electrolytes in the gastrointestinal tract in which the generated metabolites become more hydrophilic and can be re-circulated in the entero-hepatic circulation [38]. In fact, rhein, the active metabolite of acetylrhein, is the predominant form found in bloodstream that possesses potent bioactivities in mammals including rodents, rabbits and human [39]. The pharmacological actions of this anthraquinone derivative have been lately evidenced in various inflammatory diseases such as arthritis [31,40] and dermatological disorders [41]. A recent study by He et al demonstrated that rhein could effectively inhibit the activation of renal interstitial fibroblasts in mice [35] implicating its potential in targeting fibrogenesis. As the usage of rhein has been increasingly recognized, we therefore extend its usage to the pancreatic fibrogenesis in the present study.

In our *in-vivo* experiment, repetitive administration of cerulein, an analogue of the exocrine secretagogue cholecystokinin 8, aggravate acinar injury, ECM deposition and fibrosis lesion. Along with the fibrotic progression, several mechanisms including recruitment of immune cells and robust production of fibrogenic factors for tissue repair are being intensively elicited in order to protect the pancreas from autodigestion of parenchyma [42]. In the primary evaluation of severity of CP, less inflammatory infiltrates were observed in the pancreatic tissues of CP mice that were prophylactically and therapeutically fed with rhein; meanwhile, the cerulein-elevated systemic pro-inflammatory cytokines namely TNF-α and IL-1β were accordingly reduced in those rhein-treated animals. Our results here are in agreement to the findings of other research groups that the anthraquinone compound exerts pharmacological activities against inflammatory diseases majorly inhibiting the production of TNF-α and IL-1β and hence resulted in a suppressed recruitment of immune cells [31,43]. In terms of pancreatic architecture, the degree of atrophy was significantly attenuated in those rhein-treated CP mice as implicated by the substantial restoration of their pancreatic wet weights. According to some previous reports, rhein at doses in the range of 25 to 150 mg/kg were shown to exert protective effects against inflammatory diseases including arthritis and dermatological disorders within different consumption periods [44]. The present study undoubtedly demonstrated that treatments with rhein at 50 mg/kg do ameliorate the severity of CP in mice. 

Fibrogenesis and morphological scarring of the parenchymal tissues are the characteristic pathological alterations of CP in which the activation of PSCs is indispensable. In the cerulein-treated mice, activated PSCs and ECM deposition were identified by the presence of α-SMA, elevated levels of FN1 and COL I-α1 in the exocrine pancreatic tissues. A previous report by Guo and colleagues showed that rhein at a dose as low as 25 mg/kg/day provided significant protective effect against carbon tetrachloride-induced liver fibrosis in rats [36]. In the current study, we clearly demonstrate that rhein at 50 mg/kg/day significantly attenuates PSC activation and ameliorates pancreatic fibrosis in the cerulein-induced CP mice. Between the prophylactic treatment group Cer+Rh-A (6-week treatment) and the therapeutic treatment group Cer+Rh-B (3-week treatment), the longer consumption of rhein (i.e. Cer+Rh-A), probably with a preventive purpose, provides a better histopathological score of fibrotic conditions; therefore, we deduce that the levels of re-circulating metabolic rhein is important to the management of the disease. The progression of fibrotic events appears to be alleviated as the number of activated PSCs is reduced. On the other hand, it is interesting that rhein exerts suppressive effects comparable to some well-known anti-fibrotic agents such as curcumin [45] and ellagic acid [46] on PSC activation *in-vivo* and *in-vitro*.

The immortalized rat PSC line LTC-14 [37] provides us the greatest convenience in the investigation of cellular mechanisms of fibrosis. Upon the addition of recombinant TGF-β, expression levels of stress filament α-SMA as well as ECM proteins FN1 and COL I-α1 in LTC-14 cells were significantly up-regulated. The LTC-14 cells also responded to pro-inflammatory cytokine TNF-α and led to elevated expression levels of *Acta2* and *Tgf-β*; therefore, pro-inflammatory cytokines such as TNF-α do play important roles in fibrotic events. Our results are in accordance to recent reports that the levels of pro-inflammatory cytokines positively correlate to the severity of fibrosis [47,48]. At the molecular level, the repairing machinery for the injured or inflamed parenchyma creates a microenvironment that provides pro-regeneratory effects and simultaneously promotes the production of various pro-fibrotic factors in large amounts in which fibrogenesis is elicited. In our *in vivo* experiments, the obvious reduction in pancreatic *Tgf-β* and *Acta2* expressions of rhein-treated CP mice is plausibly the partial consequence of the alleviation in inflammatory responses, i.e. declined serum levels of TNF-α and IL-1β. As rhein suppresses inflammatory responses, the fibrogenesis cascade is not being robustly up-regulated upon the onset of pancreatic injury. The sequential pro-fibrotic processes are thus decelerated and resulted in a significant decrease in pro-fibrotic mediators namely fibronectin, type I collagen and α-SMA. Therefore, both our *in-vivo* and *in-vitro* data here supports the positive correlation between the level of pro-inflammatory cytokines and the level of fibrotic mediators, hence the severity of pancreatic fibrosis.

The pathogenesis of hepatic fibrosis has been relatively well studied in the last few decades as the underlying mechanism mainly involves the activation of hepatic stellate cells (HSCs). When the quiescent stellate cells trans-differentiate into myofibroblasts, net ECM deposition is largely promoted and thus fibrogenesis is driven [49]. Similar to the role of HSCs in the development of hepatic fibrosis, the mechanism of fibrosis in the pancreas has been suggested to be the consequences of the activation and proliferation of PSCs though the capability of tissue regeneration is comparatively limited in pancreas. The current study clearly show that rhein at a physiological dosage attenuates the pivotal fibrogenic mediators α-SMA and TGF-β both *in-vitro* and *in-vivo* indicating its suppressive role in PSC activation. Our results are in accordance with the study performed by Guo and colleagues in rats with carbon tetrachloride 4-induced liver fibrosis. In Guo’s case, rhein ameliorated the degree of liver fibrosis by down-regulating hepatic levels of α-SMA and TGF-β, and thus inhibiting the activation of HSCs [36]. A number of reports have shown that the activation and proliferation of PSCs positively correlate to the production of ECM proteins probably via TGF-β-related pathway [50,51]. In the present study, TGF-β stimulation significantly increases the synthesis and secretion of ECM proteins namely FN1 and type I collagen in LTC-14 cells. Our results are comparable to earlier studies done by research groups of Vogelamann [52] and Shek [53] as activated PSCs have been suggested to be the main source of collagen in CP [54]. In this study, the *Tgf-β* level in pancreatic homogenates in rhein-treated CP mice was found to be suppressed and tissue atrophy was subsequently declined. Thus, inhibition of ECM deposition by rhein definitely attributes to the amelioration of the pancreatic fibrosis and CP.

**Figure 7:**
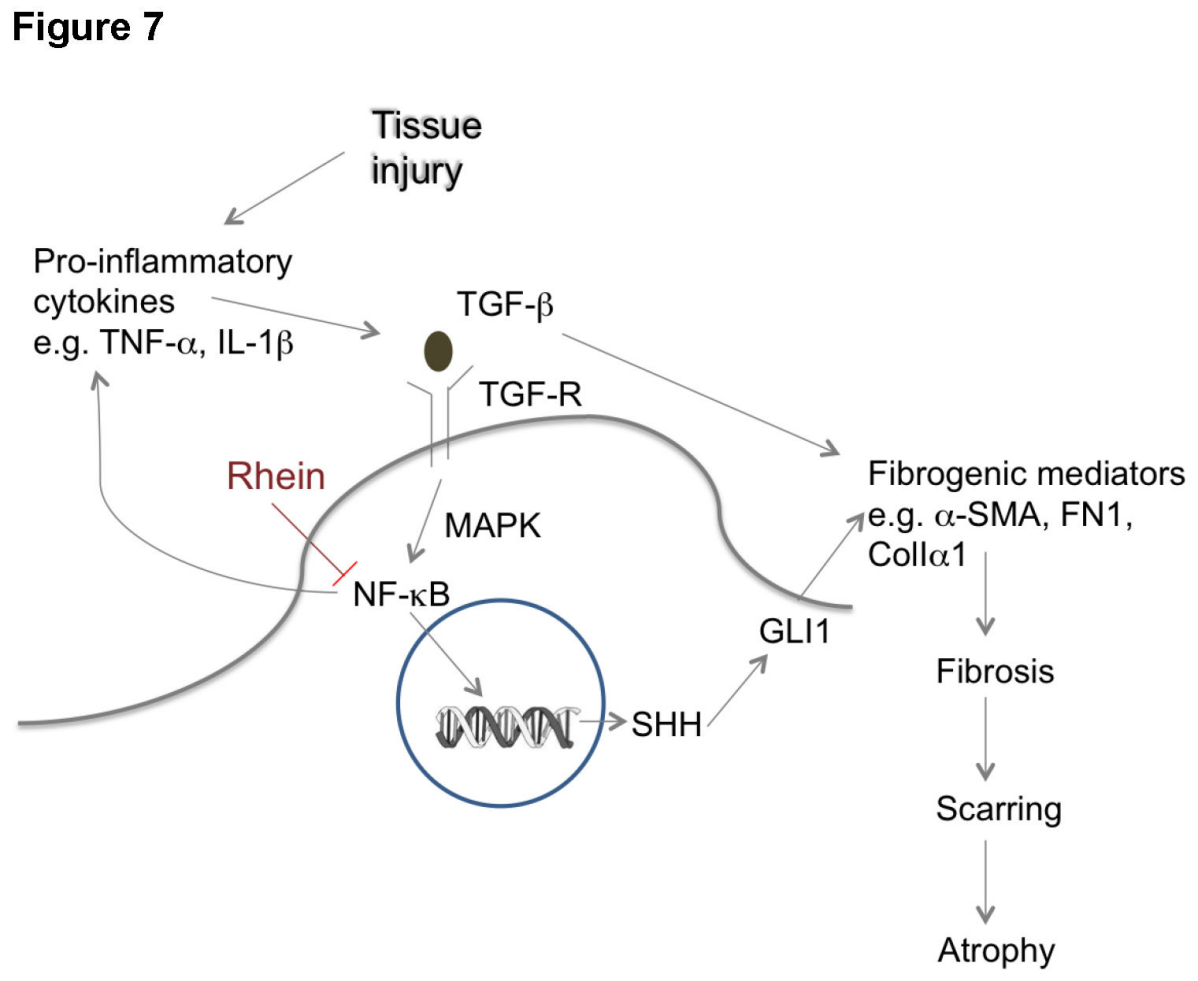
A schematic flow chart showing the roles of TGF-β and SHH/GLI1 signaling pathways in pancreatic fibrosis.

The central signaling cascade modulating the activation of PSCs and production of ECM is extremely crucial to the attenuation of fibrogenesis. Two very recent studies demonstrated that SHH is a key regulator to the initiation of fibrosis in renal [19] and pulmonary tissues [20]. From the results of our *in-vivo* study, we notice that the pancreatic SHH level was substantially up-regulated under CP conditions implicating that Shh signaling pathway is associated with the development of pancreatic inflammation and fibrosis. The elevated expression of SHH by the exogenous addition of recombinant TGF-β in LTC-14 cells further manifests the crosstalk between TGF-β signals and SHH pathway in PSC activation. The SHH signaling is suggested to be mediated through its immediate target GLI2 in hepatic fibrosis since GLI1 expression was found rather sparse in HSCs [55]. In contrast, HH effector GLI1, but not GLI2, was dramatically up-regulated under fibrotic conditions in kidney [56]. In pancreatic fibrosis, GLI1 appears to be the dominant down-stream effector of Shh as substantial increases of *Gli1* transcripts were obtained in CP pancreatic tissues in our present study. The propagation of SHH signaling differs between hepatic and pancreatic fibrogenesis although HSCs and PSCs play similar roles in the development of the respective fibrotic diseases. The initiation of the SHH signaling cascade probably involves NF-κB activation as reported by other research groups that p65 binds to Shh promoter and therefore stimulates Shh activities [57,58]. In the present study, exogenous TGF-β stimulates the trans-activation of NF-κB and the expression of SHH in cultured PSCs, and such stimulation is sufficiently inhibited by the addition of rhein at 10 μM and 100 μM. Taken together of our *in-vivo* and *in-vitro* data, we suggest that SHH/GLI1 signaling emerges as an indispensable regulator of the activation of PSCs and the production of ECM proteins in the pathogenesis of pancreatic fibrosis (Figure 7).

It is noteworthy to study the detailed mechanism of pancreatic fibrosis and CP as patients with CP have a greater risk of developing pancreatic cancer that is fatal in 95 % of cases. For targeting the disease, the present study demonstrated that the natural anthraquinone compound rhein exerts promising anti-fibrotic effects on cerulein-induced CP in mice. With the prolonged administration of rhein, the number of activated PSCs and the imbalanced production of ECM are plausibly attenuated via the suppression of SHH/GLI1 signaling pathway. Reductions in systemic pro-inflammatory cytokines and pro-fibrotic mediators by rhein positively correlate to the inhibition of progressive atrophy of pancreatic tissue and degree of fibrosis. In conclusion, we strongly suggest that rhein may serve as a therapeutic agent for the clinical management of pancreatic fibrosis and PSC-related pathologies.
